# Comparison of Dentinal Defects Formation in Straight, Moderate and Severely Curved Canals by Three Distinctive Nickel Titanium Instruments: An *in vitro* Study

**DOI:** 10.30476/dentjods.2022.94475.1787

**Published:** 2023-09

**Authors:** Azin Alasvand Javadi, Mansour Jafarzadeh, Mohammad Yazdizadeh, Neda Askari Hasanvand, Shadi Nikoo Nejad, Ali Amiri

**Affiliations:** 1 Dentist, Ahvaz, Iran; 2 Dept. of Endodontics, Faculty of Dentistry, Ahvaz Jundishapur University of Medical Sciences, Ahvaz, Iran

**Keywords:** Cracked Tooth Syndrome, Dentin, Nitinol, Root canal preparation, Root fracture

## Abstract

**Statement of the Problem:**

It is stated that engine-driven instruments might cause dentinal defects during root canal preparation. These defects might spread and progress into greater fractures or vertical root fracture.

**Purpose:**

This study aimed to compare the incidence of dentinal defects that might arise all through preparation of root canals, using One Shape, RaCe, and WaveOne systems in canals with a curvature (0-20°) and (20-40°).

**Materials and Method:**

In this *in vitro* study, 150 mandibular first molars were enrolled. Based on the degree of curvature in the mesial roots, the samples were divided into two groups (n=75) of straight and moderately curved canals (0-20º), and severely curved canals (20-40°). Then each group was randomly divided into four sub-groups. In subgroups 1 to 3 from each group, canals were prepared using WaveOne, One Shape, and RaCe. Then all roots were sectioned at 3, 6 and 9-mm distances from the apex. The slices were evaluated using stereomicroscope at 12× magnification. The data were analyzed using the Chi-square and Fisher’s exact tests and the level of significance was set at 0.05.

**Results:**

Fracture and other defects were not found in the control groups. In canals with curvature (0-20°), WaveOne caused the maximum dentinal defects and RaCe produced the least. Moreover, in canals with curvature (20-40°), One Shape caused the maximum dentinal defects while WaveOne and RaCe caused equal dentinal defects approximately.

**Conclusion:**

There was a statistically significant relationship between the performance of RaCe and One Shape in canals with curvature (0-20º) and (20-40º), (*p*Value< 0.05) while no statistically significant difference was observed in connection to the performance of WaveOne (*p*> 0.05).

## Introduction

Root canal treatment includes treating vital and necrotic dental pulps; accordingly, patients are able to preserve their natural teeth in function and esthetics [ 1 ]. Even though effective root canal therapy is determined by various elements, one of the utmost significant phases in any root canal therapy is the preparation of canal [ 1 ]. This is critical since preparation defines the effectiveness of all succeeding procedures and consists of mechanical debridement, establishment of room for medicament distribution, and enhanced canal geometries for satisfactory obturation [ 1 ]. For several years, root canal preparation was done by means of stainless steel hand endodontic files [ 2 ]. Numerous engine-driven nickel-titanium (Ni-Ti) file systems were presented for the preparation of root canals. Ni-Ti instruments offer various benefits in comparison to traditional files. Augmented flexibility and reduced operational time are the chief benefits of NiTi files [ 2 ]. Although some studies have reported that stress on the wall of root canal may increase and cause dentinal defects because of the altered cutting blade outline, taper, and tip design of these systems [ 3 - 4 ]. Onnink *et al*. [ 5 ] were the earliest to document dentinal defects as a result of canal preparation. There has been an absence of data about the distribution and features of dentinal defects according to definite standards so far. The American Association of Endodontics (AAE) categorized longitudinal tooth fractures into five main classes including craze line, fractured cusp, cracked tooth, split tooth, and vertical root fracture (VRF) [ 6 ]. Bier *et al*. [ 4 ] suggested another classification consisting of three parts including ‘no defect’, ‘fracture’, and ‘other defects’. Even if dentinal defects are recognized, it is challenging to assess the prognosis since there is no precise method to identify the progression of the dentinal defects [ 7 ]. This circumstance has always offered a restorative grip for clinicians, since a dentinal defect has a doubtful prognosis and subsequent treatment including extraction [ 8 ]. The choice to treat teeth with dentinal defects would lead to a dispute with patients about the prognosis, treatment charge, and treatment period [ 7 ]. Moreover, VRF is an imperative clinical obstacle; it is the second most common detectible cause for loss of endodontically treated teeth [ 9 ]. When VRF takes place, little can be done to resolve the current situation. Therefore, it is vital to explore elements associated with dentinal defects formation that possibly would deliver useful suggestions about prevention [ 10 ]. It is noteworthy that a vast frequency of curved root canals could be perceived in clinical practice. It is indefinite at this time if engine-driven NiTi file systems produce greater dentinal defects in severely curved canals. There are various methods to measure and classify the root canals according to the degree of curvature. Based on Schneider’s method [ 11 ], if the angle is lesser than 5°, the canal is considered straight; if the angle is 5‑20°, the canal is defined as moderately curved; and if the angle is superior than 20°, the canal is categorized as a severely curved canal.

One Shape rotary NiTi files (Micro Mega, France) with continuous rotation are made of conventional NiTi and have three dissimilar cross-section areas. The first zone demonstrates a varying 3-cutting-edge design; the second, in advance of the conversion, has a cross-section that gradually alters from 3 to 2 cutting edges and the latter (coronal) is arranged for 2 cutting edges [ 12 ]. The system is made up of one sterile single file for root canal shaping (ISO 25 tip and 6% taper) with non-working (safety) tip and variable pitch tip [ 12 ]. 

The RaCe rotary endodontic system (FKG Dentaire, La Chaux-de-Fonds, Switzerland) is created from an austenite NiTi alloy [ 13 ]. It is manifested by a triangular cross-section, with two short, sharp alternate cutting edges to be found at a different angle along the file length attributable to the alternate twisting and untwisting sections. Electrochemical polishing feature elevates its resistance to fatigue and corrosion [ 13 ]. 

The WaveOne reciprocating system (Dentsply Maillefer, Ballaigues, Switzerland) contains three sterile single-use files with non-cutting modified guiding. The WaveOne instruments have an inverse helix and two distinctive cross-sections along the longitude of their active segments. All these instruments have a convex triangular cross-sectional at the coronal termination and a modified convex triangular cross-section at the tip end. In the production process of WaveOne files, M-Wire alloys are used [ 12 ]. 

To the best of authors’ knowledge, there are no data in the literature to date, which have compared the dentinal defects formation in straight, moderate, and severely curved canals by different NiTi systems. The intention of the current study was to fill this data gap. This study was conducted to investigate the effect of three distinct NiTi systems including One Shape, RaCe and WaveOne systems on the dentinal defects formation (fracture, partial crack, and craze line) in straight and moderately curved canals(0-20º), and severely curved canals (20-40º).

## Materials and Method

**Figure 1:**
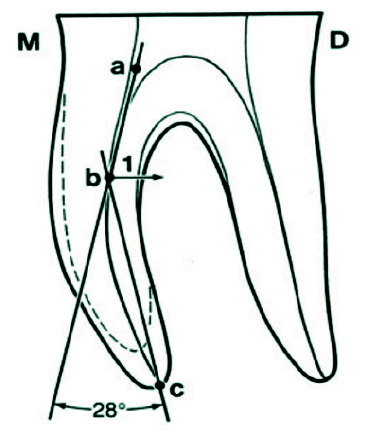
Schematic view of root canal curvature measurement according to Schneider’s method

The current study was confirmed by Ethics Committee of Jundishapur University of Medical Sciences IR.AJUMS.REC.1397.47, IR.AJUMS.REC.1395.738, and IR.AJUMS.REC.1395.14. All processes followed, were in conformity with the ethical standards of the in authority committee on human experimentation (Jundishapur University of Medical Sciences) and with the Helsinki Declaration of 1975, as revised in 2008. One hundred and fifty extracted human permanent mandibular first molars were enrolled in our study. The teeth were put in storage in purified distilled water during the course of the study. The radiographic assessment was done to omit any teeth with root resorption (external/ internal), or root defects such as craze line, partial crack, or fracture. All samples had closed apex. Then the Schneider’s method [ 11 ] was performed to define the degree of curvature of mesial root of the samples. To perform this method, after preparing the access cavity, while the file number 10 (Mani Inc., Tochigi, Japan) was inside the canal, indirect digital radiography (PSP) of all teeth was obtained from bucco-lingual dimension. The digital radiography of the teeth was performed with conventional dental radiographic unit (X genus, de Gotzen, Italy) with KVP70 and 8MA radiation for 0.16 seconds, at a distance of 2mm between the radiation source and the teeth. Exposure conditions and time were the same for all specimens, and all radiographic images were saved by Scanora software (Sordex Co., Helsinki, Finland) in TIFF (tagged image file format). All radiographs were then printed on A4 papers. First, point A was marked on the file at the cross section of the canals. A line parallel to the file was drawn from point A to the point B where the device deviates from the line. The third point (point C) was marked in the apical foramen, and then points B and C were connected in a straight line (Figure 1). The angle created between the two lines determines the amount of canal curvature. Based on the degree of curvature in the mesial root, the samples were divided into two groups (n=75); straight and moderately curved canals (0-20º) and severely curved canals (20-40°). Then each group randomly was divided into four sub-groups (three groups of twenty and one group of fifteen as a control group). The teeth were cut off atcementoenamel junction (CEJ) side by side until approximately 11 mm of the roots remains, using a high speed diamond-coated bur under abundant water coolant. The samples were inspected to approve the absenteeism of fractures/cracks under a stereomicroscope at 3× magnification (Zeiss, SV6, Jena, Germany). All roots were covered with a fine layer of silicon impression material (Coltene/Whaledent AG, Altstätten, Switzerland) to mimic the periodontal ligament (PDL) and then were inserted in acrylic blocks. To perform root canal preparation, apical patency was established using a #15 K-File (Mani Inc, Tochigi, Japan) before preparation of each canal. In the course of preparation, 12 mL per canal of sodium hypochlorite 5.25% (Cerkamed-Chloraxid5.25%, Cerkamed, Poland) was used to irrigate the canals using a 27-gauge needle. In subgroups 1 to 3 from each group, canals were prepared using WaveOne up to size 25/8, One Shape 25/6 and RaCe up to size 30/6. The teeth samples were put unprepared in control groups. RaCe and One Shape files were fixed on a handpiece connected to a torque-controlled electric motor (VDW co, Munich, Germany) and were applied in accordance with the corresponding manufacturers’ instructions for each system. Ensuing the instructions of the manufacturer, all WaveOne files were run by using the X-SMART plus endo motor (Dentsply Maillefer, Ballaigues, Switzerland), which is pre-programmed for the WaveOne reciprocating file system. Prepared roots were lastly irrigated using 2 mL of purified distilled water. All roots were cut at 3, 6 and 9-mm distances from the apex through a low-speed saw (Leica, SP1600, Wetzlar, Germany) with water coolant. The slices of each group were blindly examined for presence of dentinal defects using stereomicroscope at 12× magnification (Zeiss, SV6, Jena, Germany). Samples were assessed by two observers to assess the existence of any dentinal defects. The observers were blind to the samples grouping. In cases discrepancy between observers, the samples were re-analyzed and common consent was obtained. The results were demonstrated as the percentage and number of dentinal defects in each group. The data were evaluated using the Chi-square and Fisher’s exact tests and the level of significance was set at 0.05.

## Results

The results of the study are displayed in details in Table 1 and 2. Chi-square test was applied to compare the performance of different NiTi systems in canals with curvature (0-20º) and canals with curvature (20-40°). There was a statistically significant relationship between the performance of RaCe and One Shape files in severely curved canals(20-40º) and straight to moderate curve-d canals (0-20°) (*p*< 0.05), while no statistically significant difference was detected between the performance of WaveOne files in straight to moderately curved canals (0-20º) and severely curved canals (20-40°) (*p*> 0.05).

**Table 1:** The performance of the files in different cross sections in canals with curvature 0-20° and 20-40º

		0-20º			20-40°			*p*Value
		Intact	Other defect	Fracture	Intact	Other defect	Fracture	
One Shape	3mm	20(100%)	0(0%)	0(0%)	17(85%)	2(10%)	1(5%)	0.231
	6mm	20(100%)	0(0%)	0(0%)	12(60%)	2(10%)	6(30%)	0.003
	9mm	18(90%)	1(5%)	1(5%)	12(60%)	4(20%)	4(20%)	0.123
WaveOne	3mm	16(80%)	3(15%)	1(5%)	18(90%)	2(10%)	0(0%)	0.517
	6mm	18(90%)	2(10%)	0(0%)	17(85%)	3(15%)	0(0%)	0.633
	9mm	17(85%)	1(5%)	2(10%)	18(90%)	0(0%)	2(10%)	0.598
RaCe	3mm	20(100%)	0(0%)	0(0%)	19(95%)	0(0%)	1(5%)	0.311
	6mm	19(95%)	1(5%)	0(0%)	18(90%)	1(5%)	1(5%)	0.598
	9mm	20(100%)	0(0%)	0(0%)	16(80%)	0(0%)	4(20%)	0.035

**Table 2:** Overall comparison of the performance of the files in canals with curvature 0-20º and 20-40°

	0-20º			20-40°			*p*Value
	Intact	Other defect	Fracture	Intact	Other defect	Fracture	
One Shape	58(96.7%)	1(1.7%)	1(1.7%)	41(68.3%)	8(13.3%)	11(18.3%)	<0.001
WaveOne	51(85%)	6(10%)	3(5%)	53(88.3%)	5(83%)	2(3.4%)	0.848
RaCe	59(98.3%)	1(1.7%)	0(0%)	53(88.3%)	1(1.7%)	6(110%)	0.042

## Discussion

In the current study, the development of dentinal defects following root canal preparation by using WaveOne, RaCe and One Shape files were compared in straight to moderately curved canals (0-20º) with severely curved canals (20-40°). In canals with curvature (0-20°), WaveOne files caused the maximum fracture and other defects. This result may be attributed to the kinematic movement of WaveOne files [ 14 ]. It is reported that reciprocal motion appears to permit the continuous relief of the file while it is engaged in the internal surface of the root canal in the course of shaping procedures. Torsional and flexural stresses operating on the dentin are decreased by way of repeating clockwise and anticlockwise rotations since the anticlockwise motion unlocks the file blades and diminishes stress [ 15 ]. However, as stated by some other reports [ 16 - 17 ], reciprocating files would be more susceptible to stimulate the progress or spread of dentinal defects than regular full-sequence rotary systems. This debate defines that the preparation of root canal by using a single large-tapered reciprocating file, which cuts off extensive quantities of dentin in a short period, have a tendency to generate or intensify further dentinal defects than regular preparation, which consist of a step-by-step and more gradual mechanical enlargement [ 16 - 17 ]. In our study, the performance of the WaveOne files in canals with curvature (0-20°) approves the latter claim. However, it is reported that kinematic motion has influence on dentinal defect development [ 18 ]. RaCe files produced the tiniest number of dentinal defects in canals with curvature (0-20°). In canals with curvature (20-40°), One Shape files caused the most dentinal defects. Indeed, while performing root canal shaping using a single file, the instrument, and root canal wall are exposed to substantial stress [ 19 ], and that might be the cause for the high percentage of dentinal defects in the canals prepared using One Shape files. In canals with curvature of (20-40°), WaveOne and RaCe files almost caused dentinal defects equally (11.7%), though, the percentage of fractures created by WaveOne files was less significant than RaCe files. The reason might be the composition of WaveOne files since it is reported that endodontic instruments produced with M-wire alloy have superior flexibility than those fabricated from traditional NiTi wire [ 20 ]. Therefore, the files are likely to negotiate curved canals more efficiently as the flexibility increases. This is contributed to the lesser number of fractures caused by WaveOne and also the large numbers produced by One Shape and RaCe since the last two files are made of conventional NiTi alloys [ 21 - 22 ]. The results displayed that RaCe and One Shape instruments in severely curved canals (20-40°) caused more dentinal defects to the canals, resulting in more fracture and other defects compared to the performance of the same files in straight and moderately curved canals (0-20°). In other words, there is a statistically significant relationship between the performance of RaCe and One Shape files in severely curved (20-40°) and straight to moderately curved (0-20°) canals (*p*<0.05). Because a rise in the canal curvature increases the stress in the NiTi files, subsequently, the stress augments in the root canal. Stress concentrations in the root canal can develop canal transportation, deviation and straightening, hence, giving rise to thinner zones of dentin. Thinner dentin debilitates the root structure and raise the likelihood for dentinal defects which may result in fracture or other defects (craze line or partial crack) [ 23 ]. Besides, it is reported that the file systems applied in root canal processes undergo the maximum stress in curved root canals [ 24 ]. It is noteworthy that dentine removal does not always increase the fracture likelihood. To be more specific, the removal of stress raiser areas, for instance in buccal and lingual farthest point of ribbon shaped canals; makes it less prone to fracture [ 25 ]. WaveOne manifested almost the same results in severely curved (20-40°) and straight to moderately curved (0-20°) canals. In other words, no statistically significant difference was observed between the performance of WaveOne files in straight to moderately curved and severely curved canals (p value>0.05). In fact, WaveOne caused significant fractures and other defects, both in straight to moderately curved (0-20°) and severely curved (20-40°) canals. The reason for the high percentage of other dentinal defect and fracture by the WaveOne in particular in canals with curvature (0-20°) could be related to its greater taper (8%) compared to RaCe and One Shape (6%). The taper of preparation can be a causative element in the dentinal defects formation. The greater taper, the further root dentin is eliminated and accordingly, the possibility of introducing root dentinal defects grows [ 4 ]. It is worth mentioning that the careful instrumentation of the apical area, which is identified as the critical region for instrumentation, has long been presumed to be a key element in the cleaning and shaping process. Nonetheless, an agreement has yet to be arrived at for the finest apical preparation size. Some promote a slightly tapered canal to avoid destructive apical instrumentation [ 26 ], although some reports recommend the root canals to be shaped by 6 to 8 files with greater sizes than the primary apical binding file [ 27 ]. The result of the current study illustrated that the percentage of fractures and other defects at an interval of 9 mm from the apex was greater compared to 3 and 6 mm. The origin can be found in the study that states the utmost root stresses were essentially to be found at the most curved midroot canal wall zone [ 24 ]. It can be theorized that greater root stress concentrations would lead to further canal deviation and give rise to thinner dentin regions. Thinner dentin decreases the root structure and augments the probability for apical dentinal defects [ 28 ]. Burklein *et al*. [ 17 ] disclosed that root canal preparation with both reciprocating and rotary instruments developed dentinal defects at the apical area, and reciprocating files (Reciproc and WaveOne) created meaningfully further partial dentinal cracks than full-sequence rotary systems (Mtwo and ProTaper). The results of Burklein *et al*.’s study [ 17 ] were consistent with our study. Although in canals with curvature (20-40°), the results of two study were not favorable and this could be attributed to the degree of curvature of the canals, since they used only canals with curvature less than 5°. In the study done by Liu *et al*. [ 29 ] reciprocal files (VDWGmbH,Munich, Germany) operated in reciprocating movement initiated cracks in only 5% of teeth, however, ProTaper files and OneShape (Micro-Mega, Besancon, France) performing in continuous rotation generated cracks in 50% and 35% of teeth, in turn. The results of latter study were accordant with the results of ours in canals with curvature (20-40°), although they did not specify the exact degree of the curvature of the samples in their study. It should be noted that in the study of Liu *et al*. [ 29 ] all the canal orifices were widened using Gates Glidden no.2 ,which might have caused less significant stresses on the root canal wall. 

It is worth mentioning that stereomicroscopy, which was used in the current study, has a considerably less definition than micro computed tomography (CT) imaging to evaluate dentinal defects [ 26 ]. Moreover, the regular sectioning methods enabled the assessment of only a couple slices per tooth with the real likelihood of missing a number of earlier dentinal defects alongside the root [ 30 ]. However, with micro-CT imaging, hundreds of slices could be investigated per tooth [ 26 ]. Regarding the application of micro CT, De-Deus *et al*. [ 16 ] performed a study and concluded that there was no association between dentinal defects formation and root canal preparation when BioRaCe, Reciproc, and WaveOne systems were used. The reason for the difference in the result of our study and theirs might be related to the application of micro CT in their study. Nevertheless, a rise in temperature from the application of high-resolution micro-CT scans could lead to dehydration of the samples and subsequently the expansion of previously present dentinal defects, that might have an impact on the result of study, particularly if teeth presented with dentinal defects before canal preparation are not omitted [ 30 ]. It should be noted that the use of sodium hypochlorite (5.25%) as irrigating solution in current study might have had an impact in the results. Sodium hypochlorite is broadly suggested because of its antibacterial and organic tissue decomposition features. The suggested concentration of sodium hypochlorite differs from 0.5% to 5.25%, with no agreement on the ideal concentration. Sodium hypochlorite has unfavorable influence on physical features, for instance elastic modulus, micro hardness, and flexural strength of the dentin. The alterations in the physical features of dentine are caused by modifications in the inorganic and the organic components of the dentine [ 31 ]. The reduction in flexural strength points out that far less likely force is necessary for the cohesive bonds within dentin to break down and consequently, the probability of fracture and other defects might increase [ 32 ]. The canal preparation in our study was performed by a skillful endodontist, and the clinician’s experience can affect the result of the study [ 33 ]. The abovementioned fact is important since Adorno *et al*. [ 34 ] reported that the working length has a remarkable effect on dentinal defect initiation. Their study claims that when instrumentation is limited to the apical foramen, there is a greater possibility of creating apical dentinal defects when larger instruments are applied in comparison to when instrumentation is limited to 1 mm shorter than the apical foramen. The loss of instrumentation length is more probable in dental students or dentists with reserved experience [ 16 ]. It is worth mentioning that the age and gender of the patients from whom the samples were collected might play a role in causing dentinal defects [ 35 - 36 ]. Furthermore, the incidence of dentinal defects in aged patients is believed to be greater, which is attributed to loss of dentine elasticity and augmented stress fatigue over time [ 37 ]. In other words, the cause of fracture or other defects in some samples may be related to the aged dentin rather than the brand of NiTi system or the degree of canals curvature. Additionally, the probability of fracture or other dentinal defects in an intact tooth that was extracted for orthodontic treatment is different from that of a necrotic tooth, since the colonization of bacteria, the bacterial enzymes leakage, and host-derived matrix metalloproteinases might play a part in the breakdown of the collagen fibrils in the root dentine after clinical function [ 38 ]. Considering the substantial influence of collagen microstructure to the dentin toughening mechanisms, bacteria-induced degradation of the collagen substrate might be a major possible secondary reason of fracture tendency in endodontically treated teeth [ 36 ]. There were limitations in our study that might have had an impact on the results. The sectioning technique has a major drawback concerning the potential dentinal defects prompted by sectioning method. Nevertheless, in the current study, we believe it did not occur since there were no dentinal defects in the control group.

## Conclusion

Within the limitations of this *in vitro* study, using WaveOne in canals with curvature (0-20°) and Ra-Ce in canals with curvature (20-40º) have a tendency to cause the maximum dentinal defects. There was a statistically significant relationship between the performance of Ra-Ce and One Shape in canals with curvature (0-20º) and canals with curvature(20-40º) while no statistically significant difference was observed regarding the use of WaveOne (*p*> 0.05). Based on the result of the current study, it seems that RaCe is the safest file to be used in terms of dentinal crack formation in straight, moderate, and severely curved canals and WaveOne is the least safe.

## Acknowledgment

This study supported by Department of Endodontics, Dental School, Ahvaz Jundishapur University of Medical Sciences, Ahvaz, Iran.

## Conflict of Interest

The authors declare that they have no conflict of interest.
